# A novel, nonlethal liver biopsy procedure in an elasmobranch

**DOI:** 10.1111/avj.13432

**Published:** 2025-02-25

**Authors:** AM Hasenei, L Foyle, JL Rummer

**Affiliations:** ^1^ College of Science and Engineering James Cook University Townsville Australia; ^2^ College of Public Health, Medical and Veterinary Sciences James Cook University Townsville Australia

**Keywords:** animal ethics, animal welfare, conservation, husbandry, minimally‐invasive sampling, shark stress, surgery, tissue samples

## Abstract

Tissue sampling is essential for understanding the biology, health and conservation status of elasmobranchs (i.e., sharks and rays). Historically, these samples have been obtained through recreational and commercial fisheries or via fisheries‐independent sampling, often involving lethal methods. However, with a significant number of elasmobranch species listed as conservation concerns under IUCN standards — approximately one‐third of species are threatened with extinction — there is an urgent need for nonlethal tissue sampling techniques to optimise animal care and further conservation research. Recent advances have demonstrated nonlethal liver sampling in teleost fishes, but this has rarely been attempted in elasmobranchs. Yet, in elasmobranchs, the liver is one of the largest organs, performing critical functions such as buoyancy regulation, energy storage and metabolic processes. Here, we present a nonlethal liver biopsy procedure in an elasmobranch species, the epaulette shark (*Hemiscyllium ocellatum*). Individual sharks were wild‐collected from coastal waters of Queensland, Australia and maintained in holding facilities at James Cook University and Heron Island Research station where all procedures took place. Following surgery, the sharks made a full recovery, accepted food within 24 hours, and were monitored for 2 weeks before being released back to their original collection sites after complete healing. This study aimed to showcase these methods as a foundation for improved veterinary care and conservation science, while also advocating for the broader adoption of nonlethal sampling techniques in both research and clinical practice to promote sustainability and ethical conservation efforts.

## Introduction

Liver biopsies are a critical tool in physiological research, offering invaluable insights into a wide range of range of biological processes. In fishes, liver samples are particularly important for diagnosing and detecting diseases,^1^ evaluating pollutant accumulation,^2^ assessing nutritional health and dietary impacts,^3^ conducting stable isotope analyses^4^ and examining transcriptional plasticity.^5^ These diverse applications underscore the significance of liver tissue in understanding both the health of individual organisms and broader ecological dynamics.

Historically, liver samples in fishes have been collected from commercial and recreational fisheries specimens or from lethal procedures in fisheries‐independent sampling.^6^, ^7^ However, these techniques are undesirable when working with protected and/or high‐profile, charismatic species and raise ethical and conservation concerns.

While nonlethal liver sampling has been demonstrated in some teleost species,^4^, ^7^ there is only one documented case in an elasmobranch. This was conducted on a single individual cownose ray (*Rhinoptera bonasus*) and described in a conference proceeding.^8^ Elasmobranchs are prime candidates for nonlethal liver sampling due to their status as high‐profile, often protected species, where lethal procedures are not an option. Additionally, many elasmobranch species exhibit tonic immobility, which can simplify sampling methods. Moreover, the liver in elasmobranchs is one of the largest organs, playing essential roles in buoyancy, metabolism, ketogenesis, lipid storage and the production of yolk reserves in females.^9^, ^10^ Here, we present a novel, nonlethal liver biopsy procedure for an elasmobranch species, the epaulette shark (*Hemiscyllium ocellatum*) ‐ IUCN: Least concern, to pave the way for incorporating these methods into future research and encouraging the exploration of nonlethal methodologies.

## Methodology

Ten epaulette sharks were collected from various sites along the northeast coast of Queensland and transported back to the Marine and Aquaculture Research and Facilities Unit (MARFU) at James Cook University or University of Queensland's Heron Island Research station. Animals were maintained in 600‐l recirculating aquaria at a stocking density of no more than three individuals to a tank (0.3‐kg/100‐l). All methods and procedures were conducted under appropriate James Cook University animal ethics permissions (AE2856) as well as associated Great Barrier Reef Marine Park Authority (G23‐48386.1) and Queensland General Fisheries (260101) permits.

**Figure 1:**
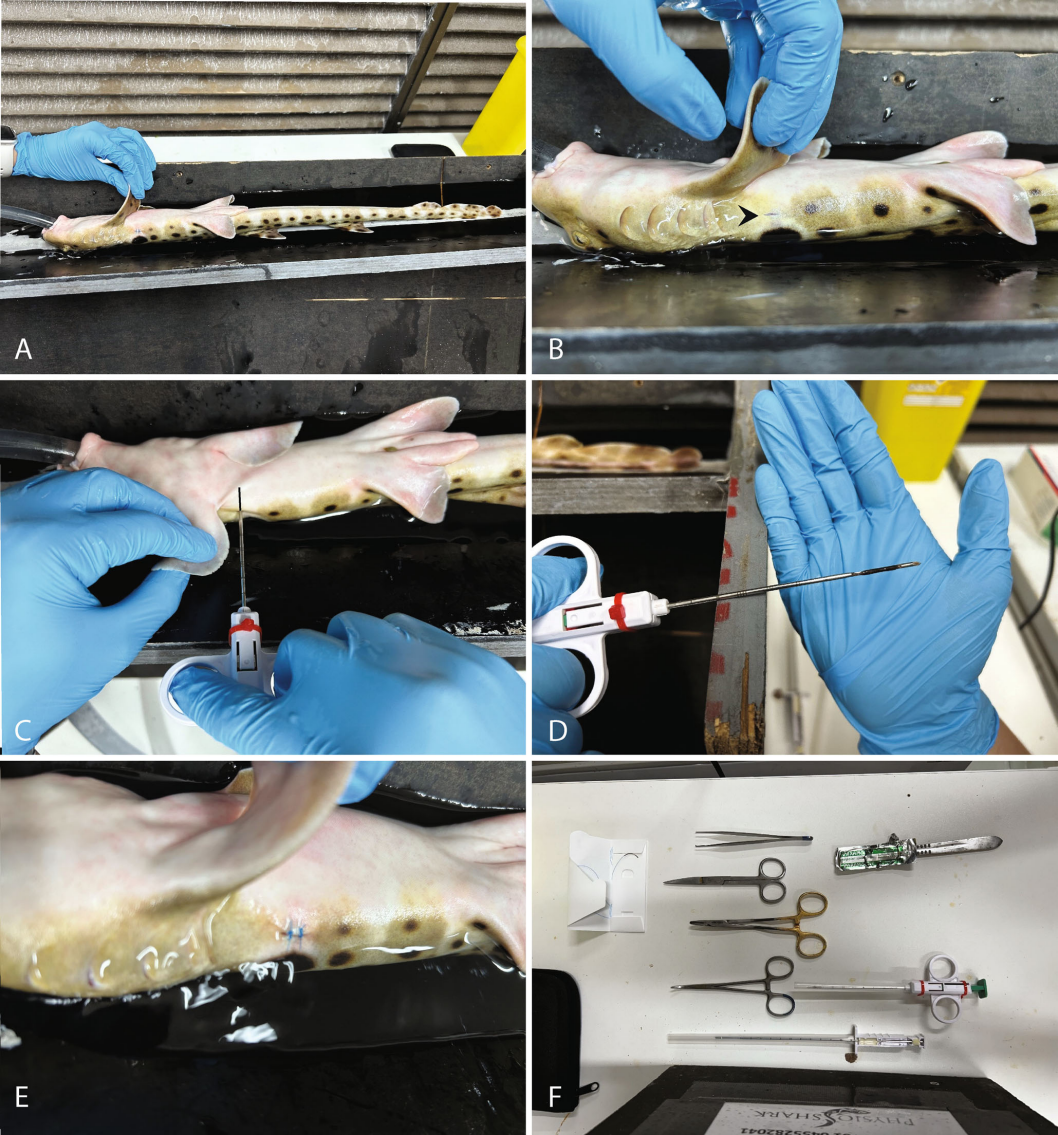
Retrieval of liver tissue via nonlethal liver biopsy procedure in *H. ocellatum*. (A) Fully sedated epaulette shark being gently ventilated with 30 mg/L of benzocaine anaesthetic. (B) An arrow indicating a 1‐centimetre incision made in an anterior–posterior direction, parallel to the dorsal plane, through the skin approximately 2 centimetres dorsal and 2 centimetres posterior to the insertion of the right pectoral fin. (C) Insertion of biopsy needle with 20‐mm needle guideline internal to the animal indicating the depth of insertion to effectively cut the liver tissue. (D) Approximately 20 mg of liver tissue removed from the animal, which was placed into RNAlater for subsequent analyses. (E) Biopsy puncture filled with an Orabase gel® and nitrofurazone mixture and the skin incision closed using two simple interrupted sutures. (F) Assortment of some of the equipment used to perform the biopsy procedure including a 14‐guage 9‐cm Tru‐Cut style biopsy needle (Covetrus®), number 22 surgical scalpel blade, forceps, needle drivers, haemostats and nylon sutures with 3/0 19‐mm reverse cutting needles (Covetrus®).

Animals were fasted for 48 hours prior to surgery. Then, a 30‐l anaesthetic bath was prepared using benzocaine dissolved in 15‐ml of 80% ethanol at a concentration of 60 mg/L and buffered to a pH of 8.2 with sodium bicarbonate.^11^ The bath was maintained according to the animal's holding tank temperatures at 28.5°C. Individuals were introduced into the anaesthetic bath and monitored for ventilation frequency, responsiveness and behavioural cues to indicate induction of level III surgical anaesthesia.^12^ Once anaesthetized, individuals were transferred to a surgical table, placed into dorsal recumbency and ventilated via a tube inserted into the mouth to pump 30 mg/L benzocaine dissolved in seawater over the gills using a submersible aquarium pump with a flow rate of 170 l/h (Figure 1A).

During the procedure, animals were constantly monitored to assess gill ventilation movements, excessive bleeding, response to tactile stimuli and gill/epithelial coloration to ensure the subject was completely anesthetized and receiving enough oxygen.^13^ Sporadic movements or neuromuscular spasms to tactile cues indicate the subject is not fully anesthetized, and a pink or purple colouration indicates an anaemic condition developed from loss of ventilatory function. Excessive bleeding has the potential to occur if the blood vessels of non‐target tissues are haemorrhaged, which can result in death of the individual.

A 1‐centimetre incision was made in an anterior–posterior direction, parallel to the dorsal plane, through the skin approximately 2 centimetres dorsal and 2 centimetres posterior to the insertion of the right pectoral fin using a number 22 surgical scalpel blade (Figure 1B). Then, a 14‐guage 9‐cm Tru‐Cut biopsy needle (Covetrus®) was inserted 90 degrees to the lateral wall of the animal to a depth of 20 mm to retrieve ~20 mg of liver tissue (Figure 1C,D). We selected to extract liver tissue from the right side of the animal, as previous dissections on cadavers revealed the right lobe of the liver to be larger in size, and the location would minimise the risk of biopsy needle‐induced trauma to non‐target tissue. Minimal bleeding, if any, occurred after engaging the biopsy needle from the sampling site and was deemed to be clinically insignificant. Concluding sampling, nitrofurazone cream mixed with Orabase gel® was applied to the surgical site, and the incision was closed using two simple interrupted nylon sutures with 3/0 19‐mm reverse cutting needles (Covetrus®) (Figure 1E). Betadine™ was applied topically to the incision site to prevent any subsequent infection.

Individuals were then transferred to a separate recovery tank and made a full recovery encompassing normal behaviours (i.e., fully upright with coordinated, routine movement) and a normalized ventilation rate between 50 and 60 Vf‐min^−1^ within 60 minutes of being removed from the anaesthetic. After 2 weeks at their original holding temperature, sutures were removed from non‐sedated animals, and once incision sites had completely healed, the sharks were released back to their original collection locations.

## Discussion

With approximately one‐third of shark species threatened with extinction and many species protected species under IUCN standards^14^ the development of nonlethal, minimally invasive techniques to assess physiological responses to anthropogenic and other stressors is critically important. Although similar methods have been successfully demonstrated in other protected species, such as vulnerable and critically endangered river sturgeon (*Scaphirhynchus sp*.), where liver samples were used to detect metal and organic chemical accumulation,^7^ our study provides the first published account of these methodologies in an elasmobranch species. Ultimately, these data can inform conservation management by assessing sublethal impacts of anthropogenic pollutants on physiological performance, gene expression and reproductive health.

Data from a nonlethally extracted liver sample may also provide insights across a range of physiological disciplines, especially when combined with other minimally‐invasive, physiological and non‐physiological tools. For example, Madliger et al.^15^ outline several, including quantifying pollutant and chemical concentrations to track population declines, assessing oxidative status in response to environmental changes, and evaluating vitellogenesis efficiency to predict reproductive output – all achievable through nonlethal liver biopsies. Integrating these findings with biotelemetry would add a spatial ecology dimension, helping to pinpoint both point and non‐point sources of pollution affecting populations. This approach could provide the necessary justification and empirical evidence to mitigate these stressors using a nonlethal procedure. A multitool, integrated approach would offer a more comprehensive understanding of how organisms respond to environmental challenges, and the versatility nonlethal liver sampling in this context cannot be overstated.^15^ Additionally, many elasmobranch species are maintained in captivity for educational or research, necessitating optimal husbandry and nutrition protocols. Nonlethal liver sampling can help assess key biomarkers, such as amino acid, lipid and fatty acid profiles, to evaluate nutritional status and inform best practices for their care.

To support future research endeavours utilising hepatic or intracoelomic biopsy in clinical or research settings, we offer key insights from our experience in designing and implementing these procedures. Due to the small‐bodied nature of the epaulette shark and its lack of tonic immobility, we opted to anaesthetise individuals to level III surgical anaesthesia prior to commencing this procedure. This approach minimised the risk of sporadic movement or neuromuscular spasms that could cause injury or lethal haemorrhage. It is essential to carefully consider whether local or general anaesthesia is appropriate and to determine the effective dosage based on the animal's morphology, physiology and the sampling environment. These techniques could be adopted for use in the field with larger‐bodied elasmobranchs, utilising induced tonic immobility, local anaesthesia and appropriate animal ethics approvals. We also recommend, when feasible, the use of a portable ultrasound and/or coelioscopy, similar to Perry et al.^8^ to visualise organ location and depth, thereby minimising the risk of trauma to non‐target tissues and optimising animal welfare. We encourage future research to prioritize refinement of nonlethal methodologies for any excision procedure across taxa after careful consideration of its use in research animals. Careful consideration of the sampling method and route, minimising tissue extraction while maximising its utility, implementing technologies to streamline procedures safely, and ensuring that sample directly benefits of the individual or species are all essential to maintaining high ethical standards.

Our ultimate goal is to refine methodologies and encourage interdisciplinary applications that minimise the need for lethal sampling techniques in species of conservation and ethical concern. Nonlethal liver sampling in both captive and wild elasmobranchs provides invaluable insights into health, nutritional status, physiological performance and conservation genetics. By advancing these techniques, we aim to support future research and aquatic animal practitioners in improving conservation strategies and husbandry practices for elasmobranchs and beyond.

## Conflicts of interest and sources of funding

The authors confirm no conflict of interest associated with this manuscript.

## Data Availability

Data sharing is not applicable to this article as no new data were created or analyzed in this study.
